# Quality of Life in Multiple Sclerosis Compared to Amyotrophic Lateral Sclerosis: Fatigue and Fast Disease Progression Interferes with the Ability to Psychosocially Adjust

**DOI:** 10.3390/brainsci15070745

**Published:** 2025-07-11

**Authors:** Luisa T. Balz, Ingo Uttner, Jochen Weishaupt, Albert C. Ludolph, Daniela Taranu, Ioannis Vardakas, Stefanie Jung, Tanja Fangerau, Deborah K. Erhart, Makbule Senel, Hayrettin Tumani, Dorothée E. Lulé

**Affiliations:** Department of Neurology, Faculty of Medicine, Ulm University, D-89081 Ulm, Germany; luisa.balz@uni-ulm.de (L.T.B.); ingo.uttner@uni-ulm.de (I.U.); jochen.weishaupt@uni-ulm.de (J.W.); albert-c.ludolph@uni-ulm.de (A.C.L.); daniela.taranu@uni-ulm.de (D.T.); ioannis.vardakas@uni-ulm.de (I.V.); stefanie.jung@rku.de (S.J.); tanja.fangerau@rku.de (T.F.); deborah.erhart@uni-ulm.de (D.K.E.); makbule.senel@uni-ulm.de (M.S.); hayrettin.tumani@uni-ulm.de (H.T.)

**Keywords:** multiple sclerosis, psychosocial adjustment, quality of life, depression, fatigue

## Abstract

**Background/Objectives**: Multiple sclerosis (MS) is a complex neurological disease that is associated with a broad spectrum of physical and psychological symptoms. Psychosocial adjustment (PSA) refers to the ability to cope with these challenges, which influence quality of life (QoL) and depressiveness in ways not yet fully understood. This study explores the relationship of PSA and disease-specific symptoms in MS, including fatigue, a prominent MS symptom. Additionally, PSA was compared to Amyotrophic Lateral Sclerosis (ALS) to disentangle the impact of disease trajectory on PSA. **Methods**: We interviewed 77 MS patients using patient-reported outcome measures on QoL and depression and compared them to 30 ALS patients. Confirmatory factor analysis and regression analysis were used to identify PSA indicators and predictors in MS, while *t*-tests assessed PSA differences across diseases. **Results**: Key PSA indicators in MS included physical (PQoL), mental (MQoL), and subjective (SQoL) quality of life, as well as depressiveness, with cognitive and motor fatigue emerging as significant predictors. MS patients had higher PQoL and SQoL and lower levels of depression compared to ALS patients, while both groups were comparable with regard to MQoL. **Conclusions**: PSA in MS is supported by high QoL and low depression levels, with fatigue being a significant predictor. Despite different disease trajectories, patients with MS and ALS showed comparable MQoL, indicating that both diseases similarly impact mental QoL, reflecting a partial overlap in psychosocial adjustment. Overall, psychosocial adjustment was more favorable in MS, likely due to its slower disease progression compared to ALS.

## 1. Introduction

Multiple sclerosis (MS) is characterized by inflammation, demyelination, and axonal degeneration, leading to a wide range of neurological symptoms such as visual disturbances, limb weakness, and gait problems [1]. Over time, the disease can cause irreversible disability, affecting multiple body systems and significantly impairing psychological, cognitive, and occupational functions [1,2]. Consequently, the diagnosis of MS is a highly stressful life event, necessitating psychosocial adjustment (PSA), which then determines patient’s wellbeing. PSA in MS patients involves adapting to significant changes caused by the disease’s severity and its extensive impact [2,3,4]. As the process of adjustment to a severe disease is complex, multi-faceted, and dynamic, defining successful PSA is challenging. One option to address PSA in the clinical context is to measure consequences of PSA, including psychological wellbeing. As there is a lack of a universal definition of PSA outcome measures, many researchers traditionally focus on quality of life (QoL) and depression in cross-sectional studies to report outcome measures of PSA in MS [2,5,6,7]. QoL is a multidimensional construct, which encompasses physical, psychological, and social dimensions, not merely physical integrity [8,9]. QoL can be broadly divided into health-related QoL—which itself includes physical and mental QoL components—and subjective QoL, which reflects an individual’s personal evaluation of life satisfaction and emotional wellbeing. Research indicates that MS patients experience lower QoL and higher levels of depression compared to other disabled populations, which may be attributed to disease-specific factors that hamper successful psychosocial adjustment in MS. Current research provides evidence that among these, psychosocial factors may provide the strongest impact on QoL and the level of depression, even more than the disease’s severity itself [3,6,10,11,12]. Yet, most research in MS—and, notably, as shown in studies on neurodegenerative diseases such as Amyotrophic Lateral Sclerosis (ALS)—focuses on health-related quality of life, which by definition interferes with disease severity [13,14]. Mental aspects of quality of life may be less interrelated with disease progression, but this interaction is so far poorly understood [15]. What has been known is the close interaction of subjective QoL and depression in MS [15]. Depression is a prevalent comorbidity in MS, with studies indicating that it affects approximately 27% of patients at any given time and up to 50% over their lifetime [6,16,17,18]. This risk is notably higher in the years immediately following diagnosis, where patients often experience elevated levels of depression and anxiety [19,20,21]. Depression in MS is not only more common than in other neurological disorders but also tends to impair both the mental and physical components of QoL [3,7,22,23,24]. The persistent nature of depression in MS highlights its significance as a major contributor to the overall burden of the disease, further exacerbating the challenges faced by patients. Fatigue is prevalent among MS patients, with reported rates ranging from 18.2% to 97% [25], it is characterized by a profound sense of tiredness and lack of energy that disrupts normal activities, distinct from feelings of sadness or weakness [26]. Fatigue has been shown to predict lower QoL and is closely associated with the severity of depressive symptoms in MS patients [1,11,22,26,27,28,29,30,31,32,33]. These findings highlight the critical role of fatigue in the overall disease burden, making it a key factor in both the psychological and physical wellbeing of MS patients. Overall, poor PSA is commonly reported in MS patients [34], and so far, little is understood about PSA in MS, especially with regard to clinical course and severity. While MS, as a chronic inflammatory disease of the central nervous system, shares some clinical features—such as movement impairment and QoL decline—with neurodegenerative diseases like ALS, its slower disease progression and specific symptoms like fatigue may uniquely affect PSA. Importantly, while PSA has already been investigated in ALS [35], it remains underexplored in MS. This study therefore focuses on describing PSA in MS and identifying fatigue as a predictor, while additionally drawing comparisons with PSA in ALS to explore how different disease trajectories shape PSA outcomes. Factors indicating successful PSA in these diseases are, among others, physical (PQoL), mental (MQoL), and subjective (SQoL) quality of life, as well as the level of depression.

## 2. Materials and Methods

### 2.1. Design and Participants

Seventy-seven MS patients and thirty ALS patients were recruited from the Department of Neurology at Ulm University and gave informed consent prior to their inclusion in the study. The study was approved by the Ethics Committee of the University of Ulm (No. 335/23, No. 381/19). We used a prospective cross-sectional survey design, with a 12-month data collection period for MS patients (2024) and 7 months for ALS patients (2022). The analyses presented in this study are post hoc and exploratory in nature. The inclusion criterion for patients was either a diagnosis of MS with clinically and laboratory definite MS according to McDonald’s criteria [36] or ALS according to the revised El-Escorial criteria [37]. Exclusion criteria were insufficient skills of the German language and physical constraints preventing task completion. Physical impairment was measured using the Expanded Disability Status Scale (EDSS with values from 0 = normal neurological function to 10 = death [38]) in MS and the ALS functional rating scale (revised form, ALS-FRS-R [39] with values from 0 = complete paralysis to 48 = normal physical function) in ALS.

### 2.2. Quality of Life and Depression as Outcome Measures of PSA

Patient-reported questionnaires were selected to assess important outcomes and indicators of PSA that are relevant to people with MS, such as subjective and health-related quality of life, and the severity of depression. SQoL was assessed with the Anamnestic Comparative Self-Assessment (ACSA; range −5 for as bad as possible and +5 as good as possible [8,40]), a widely used instrument capturing SQoL independently of physical impairments. MQoL and PQoL were assessed using the mental and physical component score (MCS and PCS; a range of 0 to 50 +/−10 points corresponds one standard deviation (SD)) of the Short-Form Survey 12 Questionnaire (SF-12), which describes health-related QoL and is commonly applied in MS research [41]. In MS, depression was measured using the Hospital Anxiety and Depression Scale—Depression (HADS-D; 7 items, a range of 0 to 21 for depression), which is specifically aimed at people with physical illnesses or physical complaints and commonly used to screen for depression in patients with MS. A cut-off ≥ 8 was used to identify patients with all degrees of depression [42]. In ALS, depression was assessed using the ALS Depression Inventory (ADI-12), an instrument specially developed for ALS patients, which takes into account their special life situation (a range of 12 to 48). A cut-off ≥ 22 was used to identify patients with all degrees of depression [43]. Due to different measures for depression, a depression rate was calculated as a percentage ((raw score/maximum achievable score on the scale) × 100) for better comparability of the values.

### 2.3. Fatigue

Fatigue was assessed using the Fatigue Scale for Motor and Cognitive Functions (FSMC), which is a diagnostic method for clarifying motor and cognitive fatigue symptoms in MS patients. The FSMC is a measure of self-assessment and comprises 20 items, which are made up of subscales for the assessment of motor and cognitive fatigue (5-point Likert scale range from 1 = ‘does not apply at all’ to 5 = ‘applies completely’; a range of 10 to 50 per subscale) [44]. The following cut-offs were used:

FSMC motor score: ≥22 mild motor fatigue, ≥27 moderate motor fatigue, and ≥32 severe motor fatigue [44];
FSMC cognition score: ≥22 mild cognitive fatigue, ≥28 moderate cognitive fatigue, and ≥34 severe cognitive fatigue [44].

### 2.4. Statistical Analysis

Statistical analyses were performed using SPSS version 29 (IBM Corp., Armonk, NY, USA) and R (lavaan package). Statistical significance was set at α < 0.05. A confirmatory factor analysis (CFA) was performed with R using the maximum likelihood method for N = 77 MS patients. The quality of the measurement model was evaluated using the Comparative Fit Index (CFI), Root Mean Square Error of Approximation (RMSEA), and Standardized Root Mean Square Residual (SRMR) criteria [45]. In addition, the factor loadings of each indicator variable (HADS-D, ACSA, MCS, and PCS of SF-12) were calculated and tested for significance, with the variance of PSA fixed at 1 to provide a metric for the latent factor. Multiple regression analyses were performed to assess the predictive relationship of cognitive and motor fatigue with all PSA indicator variables. *t*-tests for independent samples were performed to assess differences in PSA between MS and ALS. A normal distribution can be assumed due to the sample size of N ≥ 30 (N = 77 MS patients, N = 30 ALS patients) and the robustness of the *t*-test [46,47].

## 3. Results

### 3.1. Demographics and Clinical Data

Regarding the MS phenotypes, the majority of MS patients presented with the relapsing–remitting form (RRMS, 64.9%), followed by secondary progressive MS (SPMS, 20.8%) and primary progressive MS (PPMS, 14.3%). MS patients were predominantly female, younger, and had longer disease duration than ALS patients. MS Patients showed minor physical impairment according to EDSS (EDSS ≤ 3.5 indicates minor physical impairment [27]) and therefore presented with a less progressed disease stage compared to ALS, who were moderately impaired according to ALS-FRS-R (ALS-FRS-R score 34–41 indicates King’s stage 2 [48]) (Table 1).

### 3.2. Indicators of Psychosocial Adjustment in MS: CFA

There was a significant difference between the PSA model and the saturated model it was tested against (*χ^2^* = 8.29, *p* = 0.016), indicating a bad model fit, and the RMSEA (0.202) was above the limit of <0.05, which did not indicate a good model fit. The value of the SRMR (0.059) did not exceed the cut-off (SRMR < 0.08), indicating a good model fit. Further, the CFI (0.933) fell below the cut-off value (CFI > 0.95) (see Table 2).

**Figure 1:**
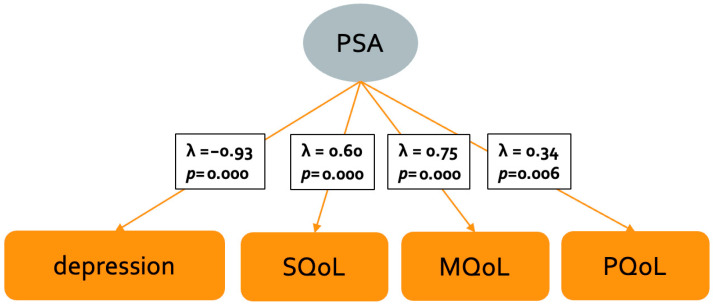
CFA model of PSA in MS. Psychosocial adjustment and its possible outcomes (orange squares). The significant indicators of PSA are highlighted in bold.

Overall, most fit indices did not show a good fitting of the PSA model, which indicates that the model needs to be adapted [45]. Despite the bad model fit, depression (λ = −0.93, *p* = 0.000) was found to be the strongest predictor of PSA, in addition to SQoL (λ = 0.60, *p* = 0.000), MQoL (λ = 0.75, *p* = 0.000), and PQoL (λ = 0.34, *p* = 0.006) (Figure 1).

### 3.3. Predictors of Psychosocial Adjustment in MS: Regression Analyses

**Figure 2:**
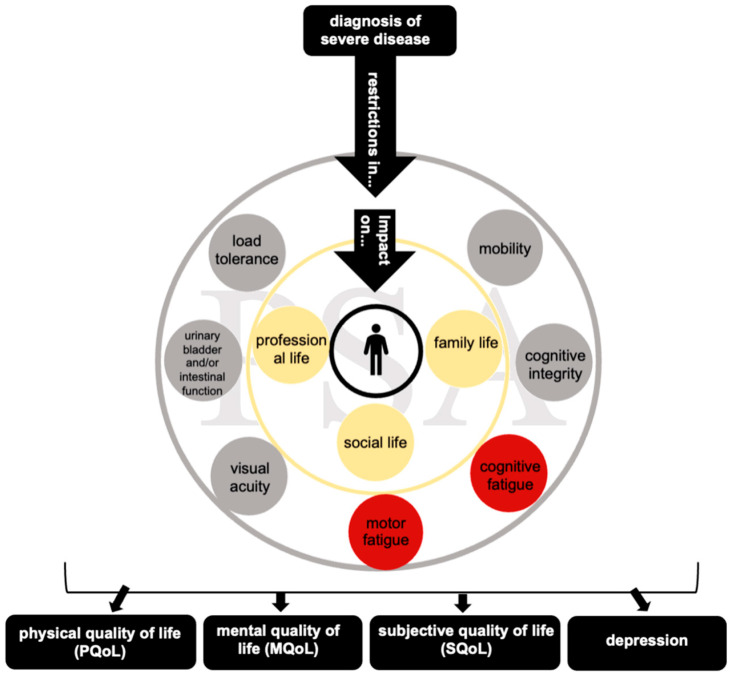
Psychosocial adjustment in MS. Model of psychosocial adjustment to a severe diagnosis with possible outcome variables (black squares). The gray/yellow rings represent the different levels patients have to adapt following a chronic diagnosis, such as MS, or a fatal neurological diagnosis, such as ALS (outer ring: internal factor; inner ring: external factors; all defined by the literature). The red circles represent potential additional predictors of PSA, which were evaluated in this study.

The Shapiro–Wilk test revealed no deviation from normality, and the assumption of homogeneity of variance was not breached. Motor fatigue was found to be a significant predictor of depression (β = 0.190, *t*(75) = 5.37, *p* < 0.001, explaining 27.8% of its variance), SQoL (β = −0.073, *t*(75) = −4.08, *p* < 0.001, explaining 18.2% of its variance), MQoL (β = −0.441, *t*(75) = −4.40, *p* < 0.001, explaining 20.5% of its variance), and PQoL (β = −0.643, *t*(75) = −8.57, *p* < 0.001, explaining 49.5% of its variance). Cognitive fatigue was identified as a significant predictor of depression (β = 0.168, *t*(75) = 4.80, *p* < 0.001, explaining 23.5% of its variance), SQoL (β = −0.059, *t*(75) = −3.30, *p* = 0.001, explaining 12.7% of its variance), MQoL (β = −0.476, *t*(75) = −5.12, *p* < 0.001, explaining 25.8% of its variance), and PQoL (β = −0.386, *t*(75) = −4.23, *p* < 0.001, explaining 19.3% of its variance) (see Figure 2).

### 3.4. Psychosocial Adjustment Comparison to ALS

A *t*-test for independent samples was performed to compare PSA (depression, SQoL, MQoL, and PQoL) in MS to ALS. The requirement for homogeneity of variance, tested by the Levene test, was not met for SQoL (*p* < 0.05), which is why the corrected *t*-test for unequal variances was calculated for SQoL. MS patients had a significantly higher SQoL (*t*(41.735) = 2.10, *p* = 0.042; d = 0.50), higher PQoL (*t*(105) = 3.64, *p* < 0.001; d = 0.78), and lower level of depression (%) (*t*(105) = −6.18, *p* < 0.001; d = −1.33) compared to ALS patients. No significant difference compared to ALS was found for MQoL (*t*(105) = −0.90, *p* = 0.373; d = −0.19) (see Table 3).

## 4. Discussion

### 4.1. Psychosocial Adjustment in MS

Our findings emphasize the complexity of PSA in MS, highlighting key factors that influence successful adaptation. Despite the challenges posed by MS, QoL measures revealed a positive outlook, with neutral-to-high scores in different QoL measures. These results suggest that many patients maintain a reasonable QoL, particularly in the subjective and mental domains. This finding may not be in line with the existing literature, as most studies have reported a moderate-to-poor QoL in MS patients but primarily focused on health-related QoL [5,7,49,50,51,52,53]. However, some QoL measures, particularly those based on eudaimonic concepts—such as physical health and economic status—often fail to capture the subjective and emotional dimensions of QoL, which are crucial for understanding PSA [5,8,29,40]. As physical health declines, these measures often indicate low QoL [49,52], as numerous studies have reported a correlation between physical function and health-related QoL in MS [3,7,22,54]. This highlights the need for PSA models to account for the subjective/hedonic aspects of wellbeing. Further, depression emerged as a central determinant, with a moderate prevalence of 26.59% in MS patients, which is consistent with previous research [6,16,17,18]. The persistent nature of depression in MS patients significantly impairs both mental and physical QoL [3,7,22,23,24], making it a critical issue that requires urgent attention in clinical practice.

The poor model fit in the CFA suggests that the PSA model in MS may benefit from refinement, potentially by integrating resilience, social support, self-esteem, and self-efficacy to better understand patient adjustment [55,56,57,58].

Fatigue, both motor and cognitive, played a key role in PSA, aligning with previous findings [2]. Many MS patients experienced severe motor and cognitive fatigue, highlighting its significant burden. Motor fatigue was closely linked to high levels of depression and a reduced quality of life, particularly in the physical domain. Cognitive fatigue showed similar effects, contributing to emotional distress and overall lower wellbeing. These findings suggest that cognitive fatigue plays a notable role in affecting not only mood (depression) but also overall QoL, with its strongest effect observed in mental QoL. The lower explained variance in subjective QoL (12.7%) implies that cognitive fatigue might have a more indirect or less pronounced effect compared to physical and mental components. In summary, these findings underscore the dual burden of fatigue in MS—as reported in previous studies—where both motor and cognitive aspects contribute to worse psychosocial outcomes [10,25,26,29,31,59], further complicating the adjustment process. Addressing fatigue in clinical practice is therefore essential for improving PSA and overall QoL in MS patients.

Importantly, our sample of MS patients included all phenotypes (PPMS, SPMS, and RRMS) without stratification, with a predominance of RRMS. Psychosocial adjustment may therefore differ in progressive subtypes, and future research should aim to examine these phenotypes separately to capture course-specific patterns more accurately. Importantly, patients with SPMS and patients with later stages of PPMS often experience more significant neurological and neurocognitive deficits, which are likely correlated with greater challenges in psychosocial adjustment.

### 4.2. Psychosocial Adjustment Compared to ALS

Despite distinct differences in etiology, clinical progression, and fatality between MS and ALS (one is fatal, the other is not), our findings suggest a notable convergence in PSA outcomes, particularly in terms of mental QoL. While the specific trajectory and symptoms of each disease vary, the psychological and emotional burdens they impose are similar, requiring comparable approaches to psychosocial adaptation. In examining single parameters of PSA, patients with MS and ALS displayed comparable mental QoL and maintained a neutral-to-positive subjective QoL. Notably, MS patients reported nearly half the levels of depression compared to ALS patients, which indicates a more favorable PSA outcome in MS patients, despite differences in disease stages and trajectories. This may be partly attributed to the moderate disease progression and the wide range of available disease-modifying treatments in MS, compared to the rapid decline seen in ALS and the limited number of treatment options, for which evidence for efficacy is very much limited [60,61]. Furthermore, the high progression rate in ALS, marked by significantly lower physical QoL, is known to disrupt PSA to a greater extent, highlighting the added challenge faced by ALS patients [13,14,35]. The lack of significant differences in mental QoL between MS and ALS suggests that both groups face similar mental health challenges, likely stemming from the chronic and incurable nature of their conditions.

### 4.3. Limitations

This study has several important limitations that should be acknowledged. First, the CFA indicated a poor model fit, as reflected by an elevated RMSEA and suboptimal CFI values, suggesting that the latent construct of perceived psychosocial adjustment may not be adequately captured by the current indicator set. Second, depression was assessed using non-equivalent instruments across diagnostic groups—HADS-D for MS and ADI-12 for ALS—introducing possible measurement bias despite normalization efforts. This compromises the comparability of depressive symptom scores and may affect interpretation of associations with PSA. Third, the cross-sectional design inherently limits the ability to infer causal relationships. While associations between fatigue, depression, and PSA are evident, temporal directionality remains unclear. Longitudinal data would be essential to elucidate dynamic changes and potential causal pathways. Finally, the study did not account for cognitive functioning, a particularly relevant omission given that cognitive impairment affects up to 40–70% of patients with multiple sclerosis [62,63,64]. Without cognitive metrics, interpretations of PSA across groups remain incomplete.

## 5. Conclusions

Our study offers valuable insights into the psychosocial adjustment of patients with MS compared to ALS. It is important to emphasize that psychosocial adjustment is an ongoing and dynamic process. In this study, depression and quality of life were used as outcome measures to indicate PSA in a cross-sectional study. With this, only a condition report of a highly complex process is presented. For future studies, longitudinal and multi-faceted measures of PSA in these severe conditions (MS and ALS) are needed to provide a deep understanding of the full scope of PSA over time. Our research significantly contributes to the existing understanding of coping with the emotional, psychological, and social consequences of chronic illness with a more comprehensive understanding of the challenges each group faces. MS patients face some unique challenges, including a high prevalence of depression and its pervasive impact of fatigue on quality of life in this population. Unlike ALS, where rapid disease progression severely impacts PSA, MS patients experience a longer, less predictable course, with fatigue emerging as a dominant factor. This adds complexity to the adjustment process, further underscoring the need for tailored interventions. Integrating mindfulness-based interventions and resilience training into care plans can be particularly effective, helping patients maintain a good quality of life [4,65,66,67,68] even in the face of severe physical challenges.

## Figures and Tables

**Table 1:** Demographics and clinical data of MS patients compared to ALS.

Characteristics	MS Patients (N = 77)		ALS Patients (N = 30)		Statistics	
	Mean (SD)	N (%)	Mean (SD)	N (%)		
Age (years)	46.83 (13.64)		56.60 (11.45)		*t*(105) = −3.47, *p* < 0.001	
Female/Male		45/32 (58.4/41.6)		10/20 (33.3/66.7)	*χ*^2^(1) = 5.45, *p* = 0.020	
Years of education	14.50 (2.82)		14.60 (3.33)		*t*(105) = −0.16, *p* = 0.876	
EDSS *	2.90 (1.84)					
ALS-FRS-R *			35.97 (9.02)			
Time since onset (years)	10.34 (9.30)		1.62 (0.87)		*t*(104) = 5.11, *p* < 0.001	
PPMS *		11 (14.3)				
SPMS *		16 (20.8)				
RRMS *		50 (64.9)				

* EDSS = Expanded Disability Status Scale; ALS-FRS-R = ALS Functional Rating Scale—Revised; PPMS = primary progressive multiple sclerosis; SPMS = secondary progressive multiple sclerosis; RRMS = relapsing–remitting multiple sclerosis.

**Table 2:** Confirmatory factor analysis (CFA) model fit indices and cut-off criteria.

Fit Index	Observed Value	Cut-off Criterion	Interpretation
*χ*^2^ *	8.29, *p* = 0.016	*p* > 0.05	poor fit (interpretation based on *p*-value)
RMSEA *	0.202	<0.05 (good), <0.08 (acceptable)	poor fit (interpretation based on index value)
SRMR *	0.059	<0.08	good fit (interpretation based on index value)
CFI *	0.933	>0.95	marginal fit (interpretation based on index value)

* *χ*^2^ = Chi-squared; RMSEA = Root Mean Square Error of Approximation; SRMR = Standardized Root Mean Square Residual; CFI = Comparative Fit Index.

**Table 3:** Determinants of psychosocial adjustment in MS and ALS.

Patient-Reported Outcome Measures	MS Patients (N = 77)		ALS Patients (N = 30)
	Mean (SD)	N (%)	Mean (SD)
HADS-D *	5.58 (4.21)		
ADI-12 *			24.97 (7.91)
Level of depression (%)	26.59 (20.05)		52.01 (16.48)
ACSA *	0.75 (2.01)		−0.40 (2.74)
SF-12 MCS *	45.79 (11.36)		47.98 (11.41)
SF-12 PCS *	42.64 (10.67)		33.74 (13.01)
FSMC motor *	30.60 (11.67)		
mild (≥22)		9 (11.7)	
moderate (≥27)		10 (13)	
severe (≥32)		38 (49.4)	
FSMC cognition *	29.05 (12.12)		
mild (≥22)		7 (9.1)	
moderate (≥28)		14 (18.2)	
severe (≥34)		31 (40.3)	

* HADS-D = Hospital Anxiety and Depression Scale—Depression; ADI-12 = ALS Depression Inventory; ACSA = Anamnestic Comparative Self-Assessment; SF-12 = Short-Form Survey 12 Items; SF-12 MCS = mental component score; SF-12 PCS = physical component score; FSMC = Fatigue Scale for Motor and Cognitive Functions.

## Data Availability

The raw data supporting the conclusions of this article will be made available by the authors on request.

## Abbreviations

The following abbreviations are used in this manuscript:

MS	Multiple sclerosis
ALS	Amyotrophic Lateral Sclerosis
PSA	Psychosocial adjustment
QoL	Quality of life
PQoL	Physical quality of life
MQoL	Mental quality of life
SQoL	Subjective quality of life
EDSS	Expanded Disability Status Scale
RRMS	Relapsing–remitting multiple sclerosis
PPMS	Primary progressive multiple sclerosis
SPMS	Secondary progressive multiple sclerosis
ALS-FRS-R	ALS Functional Rating Scale—Revised
ACSA	Anamnestic Comparative Self-Assessment
SF-12	Short-Form Survey 12 Questionnaire
MCS	Mental component score
PCS	Physical component score
HADS-D	Hospital Anxiety and Depression Scale—Depression
ADI-12	ALS Depression Inventory
FSMC	Fatigue Scale for Motor and Cognitive Functions
SD	Standard deviation
CFA	Confirmatory factor analysis
CFI	Comparative Fit Index
SRMR	Standardized Root Mean Square Residual
RMSEA	Root Mean Square Error of Approximation
